# Oncology Clinical Trials in Africa: Emerging and Operational Issues

**DOI:** 10.1200/GO.20.00233

**Published:** 2020-07-02

**Authors:** Folakemi T. Odedina, Ophira Ginsburg

**Affiliations:** ^1^ Department of Pharmacotherapy and Translational Research, University of Florida, Gainesville, FL; ^2^ New York University Langone Health, New York, NY

“Although it is the ‘gold standard’ to develop therapeutic interventions, clinical trials are not required to be conducted locally to introduce new therapies in most African countries. This is unfortunate as **optimal prevention, diagnosis and treatment decisions cannot be made for Africans** without sufficient representation of Africans in clinical trials.” —Folakemi T. Odedina, Global Congress on Oncology Clinical Trials in Blacks, 2018

Africa, especially sub-Saharan Africa (SSA), is experiencing the public health challenges of dealing with both communicable and noncommunicable diseases (NCDs), with cancer being one of the major NCDs.^1^ By 2030, deaths resulting from cancer in SSA will increase > 85%.^2^ It is also disheartening to see how African regions compare with the rest of the world in terms of the projected rise in cancer incidence and mortality from 2018 to 2040 (Figs 1 and 2).^3^ Demographic changes are a large part but do not completely explain the disproportionate increase compared with North America, in both incidence (difference, 46%) and mortality (difference, 59%) . Other factors include rising rates of tobacco use and harmful use of alcohol, in addition to other so-called commercial determinants of health that drive the risks of many cancers as well as other common NCDs.

**FIG 1:**
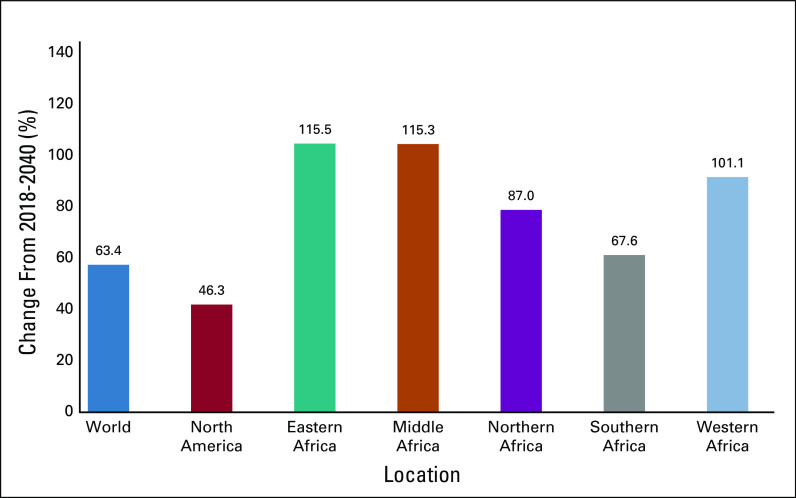
Demographic changes in cancer incidence from 2018 to 2040 for all cancers, sexes, and ages.

**FIG 2:**
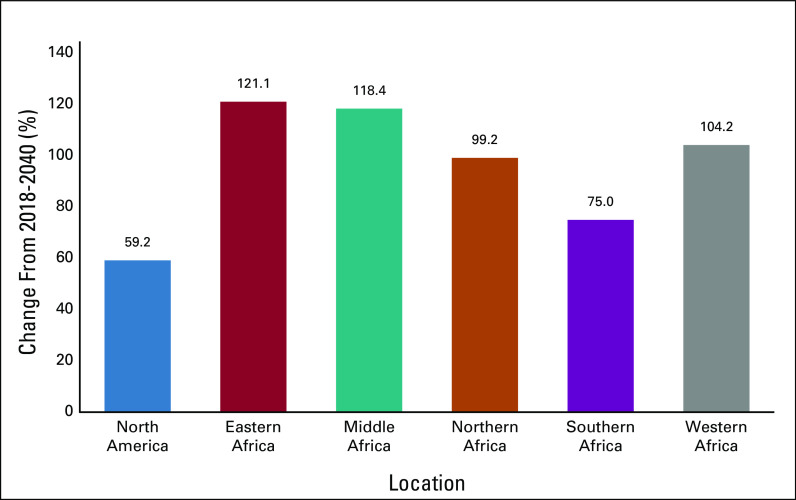
Demographic changes in cancer mortality from 2018 to 2040 for all cancers, sexes, and ages.

Without a doubt, innovative biomedical research (including clinical trials) is needed to better understand and effectively address cancer in Africa. Of urgent need are studies focused on cancer-related interventions and evaluations of the effect of such interventions on patient-related outcomes. This includes mechanistic, exploratory, feasibility, and behavioral trials in addition to traditional intervention trials. Unfortunately, there is insufficient representation of people of African ancestry in oncology clinical trials globally, thereby limiting optimal cancer prevention, diagnosis, and treatment decisions in this group.^4-7^ Africans are especially underrepresented in clinical trials.

To foster oncology clinical trials in Africa, we are releasing two Special Series. This is the first Special Series, which focuses on discussing emerging and operational issues affecting oncology clinical trials in Africa. It comprises articles from leading experts within and outside Africa covering two main domains: emerging and operational issues.

## Emerging Issues

One of the emerging critical issues with respect to oncology clinical trials in Africa involves the characteristics of current clinical trials in Africa. For example, what are the public and private registries for oncology clinical trials in Africa? Who are the sponsors of oncology clinical trials in Africa? What are the common cancer types included oncology clinical trials in Africa? These are the questions that Odedina et al^8^ answer in this series. A quantitative, Web-based, retrospective review of clinical trials registries was conducted to develop the landscape of oncology clinical trials in Africa. The authors found 109 open oncology clinical trials from several registries.^9-12^ Egypt had the most oncology clinical trials and the highest number of sponsor institutions. Most of the trials were studies of breast cancer, and the top sponsors of oncology clinical trials were academic institutions.

Ezeani et al^13^ report emerging issues identified during a town hall meeting held during the 2018 Inaugural Global Oncology Clinical Trials in Blacks conference in Lagos, Nigeria. Participants in the town hall included researchers, scientists, and advocates who contributed to a SWOT (strengths, weaknesses, opportunities, and strengths) analysis of oncology clinical trials in Africa. SWOT factors identified included improved political commitment, multidisciplinary and interdisciplinary collaborations, funding, and infrastructure.

## Operational Issues

Clinical trials cannot take place without patients or participants. Therefore, engaging patients is a critical operational issue for clinical trials in Africa. Mutebi et al^14^ discusses patient-centered approaches to the engagement and recruitment of patients for clinical trials. The involvement of patients brings “unique lived expertise and value”^14^ to the clinical trials enterprise, and it is essential to ensure their representation as research advocates. The opportunity for mobile health and clinical technology to transform oncology clinical trials in Africa is mapped out by Mutebi et al.^15^ The authors, however, caution that appropriate use must be ensured and that application context, impact, and cost effectiveness must be evaluated.

Another key operational issue for clinical trials is developing the right alliances. Solarin et al^16^ discuss the key stakeholders involved in the clinical trials enterprise, including regulatory agencies, clinicians, researchers, sites, patients, and sponsors. Fostering appropriate relationships among these stakeholders will enhance both the quality and quantity of trials in Africa.

One of the primary challenges of conducting clinical trials in Africa and a foundational operational issue involves the ethical and regulatory aspects of human research. Salhia and Olaiya^17^ discuss the ethical principles of autonomy, beneficence, and justice, answering the following important questions: What were the circumstances that led to modern-day research ethics boards? Do patients with cancer in Africa have access to clinical trials, and what are the existing oversights to protect the welfare of clinical trial participants in countries with lim-ited resources?

This series concludes with a discussion of the operational strategies for clinical trials in Africa by Graef et al.^18^ The authors present African-driven practice model examples for 10 recommendations developed during the First All Africa Clinical Trial Summit and the Operational Strategy for Clinical Trials in Nigeria Summit. The recommendations focus on funding, regulation, capacity building, and an Africa-centric patient-centered approach.

We are currently in the middle of the global COVID-19 pandemic. With > 4.5 million confirmed cases and > 300,000 deaths worldwide as of May 2020,^19^ a timely and coordinated global response to the pandemic is critical. Clinical trials have been front and center in combating the COVID-19 pandemic, which further exposes the vulnerability of most African countries with respect to preparedness for clinical trials. Furthermore, it has revealed the threats of so-called guinea pig syndrome from scientists outside Africa, with French physicians proposing to test a COVID-19 vaccine in Africa, although there is lower incidence in African countries compared with France.^18^ It is clear that African investigators need to take the lead on clinical trials in Africa while collaborating with other stakeholders within and outside Africa. We hope that this series will educate, prepare, and empower African investigators and other stakeholders toward enhancing clinical trials in Africa. Readers can expect to learn even more about the current landscape of clinical trials in Africa in another special issue of *JCO Global Oncology* next year.
